# Supplementary material for: Identification of photoperiod-induced specific miRNAs in the adrenal glands of Sunite sheep (Ovis aries)
Source: Front Vet Sci. 2022 Jul 22;9:888207. doi: 10.3389/fvets.2022.888207 (PMC9354845; doi:10.3389/fvets.2022.888207)
Supplement: Supplementary file 1 [file Data_Sheet_1.ZIP › Supplementary file/miRDeep2 file.zip!/miRDeep2 file/Know_Structure/SP42A1/oar-let-7i.pdf]

miRBase precursor : oar-let-7i
Total read count : 270068
oar-let-7i read count : 269935
remaining reads : 133

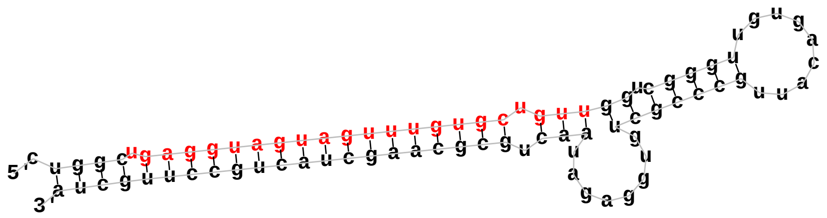

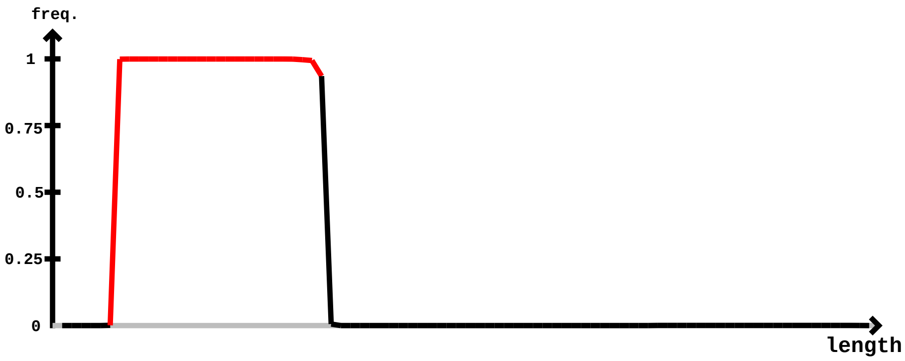


```
        oar-let-7i
5'- cuggcugagguaguaguuugugcuguuggucggguugugacauugcccgcuguggagauaacugcgcaagcuacugccuugcua   -3'    exp
    .((((.(((((((((((((((((.(((((.(((((.........)))))))........))).)))))))))))))))))))))    reads      mm      sample
    ....cugagguaguaguuugugcug...........................................................    1          0       seq
    ....cugagguaguaguuugugcugu..........................................................    18         0       seq
    ....cugagguaguaguuugugcuguu.........................................................    241        0       seq
    ....cugagguaguaguuugugcuguug........................................................    2          0       seq
    .....ugagguaguaguuugug..............................................................    5          0       seq
    .....ugUgguaguaguuugugc.............................................................    2          1       seq
    .....ugagguaguaguuugugA.............................................................    7          1       seq
    .....ugagguaguaguuugugG.............................................................    1          1       seq
    .....ugagguaguaguuugugc.............................................................    55         0       seq
    .....ugagguaguaguuGgugcu............................................................    1          1       seq
    .....ugagguaguaguuugugcu............................................................    633        0       seq
    .....ugagguaguaguuugugcug...........................................................    798        0       seq
    .....ugagguaguaguuugugcugu..........................................................    15785      0       seq
    .....ugagguaguaguuugugcuguu.........................................................    250721     0       seq
    .....ugagguaguaguuugugcuguug........................................................    1520       0       seq
    .....ugagguaguaguuugugcuguugg.......................................................    5          0       seq
    .....ugagguaguaguuugugcuguuggu......................................................    1          0       seq
    ......gagguaguaguuugugcug...........................................................    1          0       seq
    ......gagguaguaguuugugcugu..........................................................    5          0       seq
    ......gagguaguaguuugugcuguu.........................................................    128        0       seq
    .......agguaguaguuugugcuguu.........................................................    4          0       seq
    ..........uaguaguuugugcuguu.........................................................    1          0       seq
    ...........................ggucggguugugacauugcccgcuguggagauaa.......................    1          0       seq
    .............................................................cugcgcaagcuacugccuug...    1          0       seq
    .............................................................cugcgcaagcuacugccuugc..    49         0       seq
    .............................................................cugcgcaagcuacugccuugcu.    82         0       seq
```
